# Label-free characterization of ultra violet-radiation-induced changes in skin fibroblasts with Raman spectroscopy and quantitative phase microscopy

**DOI:** 10.1038/s41598-017-11091-6

**Published:** 2017-09-07

**Authors:** S. P. Singh, Sungsam Kang, Jeon Woong Kang, Peter T. C. So, Ramanchandra Rao Dasari, Zahid Yaqoob, Ishan Barman

**Affiliations:** 1 Laser Biomedical Research Center, G. R. Harrison Spectroscopy Laboratory, Massachusetts Institute of Technology, Cambridge, Massachusetts, 02139 USA; 2 Department of Mechanical Engineering, Massachusetts Institute of Technology, Cambridge, Massachusetts, 02139 USA; 3 Department of Biological Engineering, Massachusetts Institute of Technology, Cambridge, Massachusetts, 02139 USA; 4 Department of Mechanical Engineering, Johns Hopkins University, Baltimore, Maryland 21218 USA; 5 Department of Oncology, Johns Hopkins University, Baltimore, Maryland 21287 USA

## Abstract

Minimizing morbidities and mortalities associated with skin cancers requires sustained research with the goal of obtaining fresh insights into disease onset and progression under specific stimuli, particularly the influence of ultraviolet rays. In the present study, label-free profiling of skin fibroblasts exposed to time-bound ultra-violet radiation has been performed using quantitative phase imaging and Raman spectroscopy. Statistically significant differences in quantifiable biophysical parameters, such as matter density and cell dry mass, were observed with phase imaging. Accurate estimation of changes in the biochemical constituents, notably nucleic acids and proteins, was demonstrated through a combination of Raman spectroscopy and multivariate analysis of spectral patterns. Overall, the findings of this study demonstrate the promise of these non-perturbative optical modalities in accurately identifying cellular phenotypes and responses to external stimuli by combining molecular and biophysical information.

## Introduction

Quantitative, label-free profiling of cellular phenotypes, particularly during malignant transformation, remains an outstanding challenge in biomedical imaging. Changes in the cellular homeostasis in response to a stimuli, disease or therapeutic intervention are multifaceted in nature, and cannot be grasped by routinely employed targeted imaging that focuses on a small set of suspected molecules or genes. Novel approaches relying on global analysis of cellular features, from morphology to the composite biomolecular status (notably chemical composition and molecular conformation), is a pre-requisite for accurate monitoring of cellular processes. In this milieu, Raman spectroscopy (RS) and Quantitative Phase Imaging (QPI) have emerged as powerful non-destructive tools for studying cellular pathophysiology, albeit through markedly different light scattering principles^1–5^. As both methods do not require any external labels, they can be easily adapted for studying live cells in a complementary manner.

RS is a vibrational spectroscopic technique based on inelastic scattering of photons. It has been increasingly employed for molecular fingerprinting and has been incorporated as an analytical tool in the pharmaceutical and food industry^6^. In biomedical research, RS has been successfully applied for diagnosis of metabolic disorders and breast and cervical malignancies, among others^7–12^. For skin malignancies such as basal cell carcinomas (BCC), melanoma and squamous cell carcinomas (SCC), *in vivo* studies using portable clinical Raman systems has been performed^13^. Hata *et al*. have shown that Raman bands related with carotenoid concentration can be harnessed as discriminatory parameters between BCC and normal skin^14^. Other spectral features primarily related to collagen degradation (amide III), cellular proliferation (nucleic acids), water content and protein oxidation (disulphide linkages) have also been proposed as markers of different skin malignancies^15, 16^. Label-free cellular profiling using RS has been recently employed for monitoring of drug effects, senescence, heterogeneity, electroporation and various other processes^2, 4, 12, 17–20^. QPI works on principle of interferometry and measures the optical field (with phase and amplitude). As most of the biological specimens are transparent, an imaging contrast is generated by an optical phase delay, which is generally more than two orders of magnitude higher than bright field imaging^21^. Structural and biophysical profiling with QPI has been successfully employed for studying red blood cells, cardiomyocytes, neuronal cells, quality control and stem cell research^5, 21–23^.

Long-term exposure to the sun has been linked to various types of skin abnormalities including sun burn, photoimmuno suppression, photoaging, solar keratosis and photocarcinogenesis. Tumors produced by exposure to ultraviolet radiation (UVR) exposure are believed to constitute around 50% of all cancer diagnosed in USA^24^. Almost 90% of all the reported skin cancer cases are due to UVR exposure^25^. The solar radiation spectrum that reaches the earth’s surface consists of UVB (280–320 nm), UVA (320–400 nm), visible (400–800 nm) and infrared (>800 nm) rays. Differential impact of specific wavelength ranges in causing skin lesions with UVR having a singular influence in initiation of malignancies^26^. The clinical effects of UVR (which is constituted by UVA and UVB), both acute and long-term, are underpinned by multiple molecular and cellular events^27, 28^. It is generally hypothesized that initiation of skin pathologies due to prolonged exposure to sun is caused by formation of free radicals, reactive oxygen and nitrogen species (ROS) and mobilization of transition metal ions leading to impairment of DNA damage repair pathways^27, 29^. Estimation of DNA damage, glutathione, lipid peroxidation levels utilizing western blotting, flow-cytometry, antibody-coupled fluorescence imaging are routinely performed to demonstrate the influence of UVR on skin cells. However, as mentioned, the cellular alterations are multifaceted in nature and most existing tools focus on a single facet (for instance, a specific protein) that cannot really provide a comprehensive map of overall changes particularly in live, intact cell specimen.

An accurate understanding of UVR-induced changes in skin can offer new routes for recognition of cell types and the microenvironment for objective lesions detection and grading, while also facilitating the development of better treatment strategies in a patient-specific manner. In the present study, we have used QPI and RS as complementary, live cell analysis tools to evaluate the efficacy in identifying changes induced by minimal time bound UVR exposure in skin fibroblasts. Combination of these two techniques, one suited for detection of subtle morphological/biophysical alterations while the other appropriate for capturing molecular perturbations, could pave the way to address issues of label-free monitoring of cellular responses in response to an external stimulus.

## Results

### Survival Analysis

**Figure 1:**
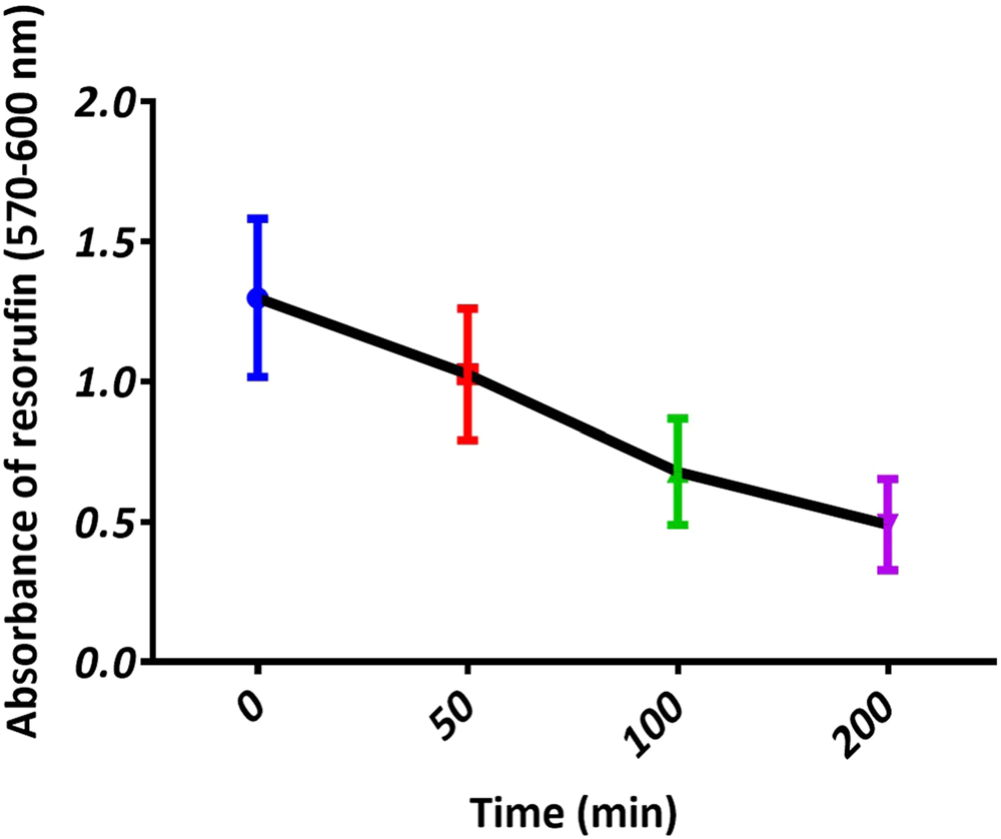
Cell survival analysis. Absorbance of resorufin dye is indicative of cell viability.

In the present study we performed the resazurin reduction test to evaluate cytotoxicity induced by UVR^30^. This test uses a dye, resazurin, to measure the biological activity of cell indirectly. Resazurin (absorption maxima at 605 nm) is reduced inside the cell with the help of mitochondrial, cytosolic and microsomal redox enzymes. The reduced dye product, resorufin, has pink colour and shifted absorption maxima (573 nm)^30^. This product can leach in to the surrounding medium and can be measured by absorbance or fluorescence spectroscopy. As shown in Fig. 1, reduction in cellular viability as a function of exposure time was observed.

### Raman spectra and multivariate analysis

**Figure 2:**
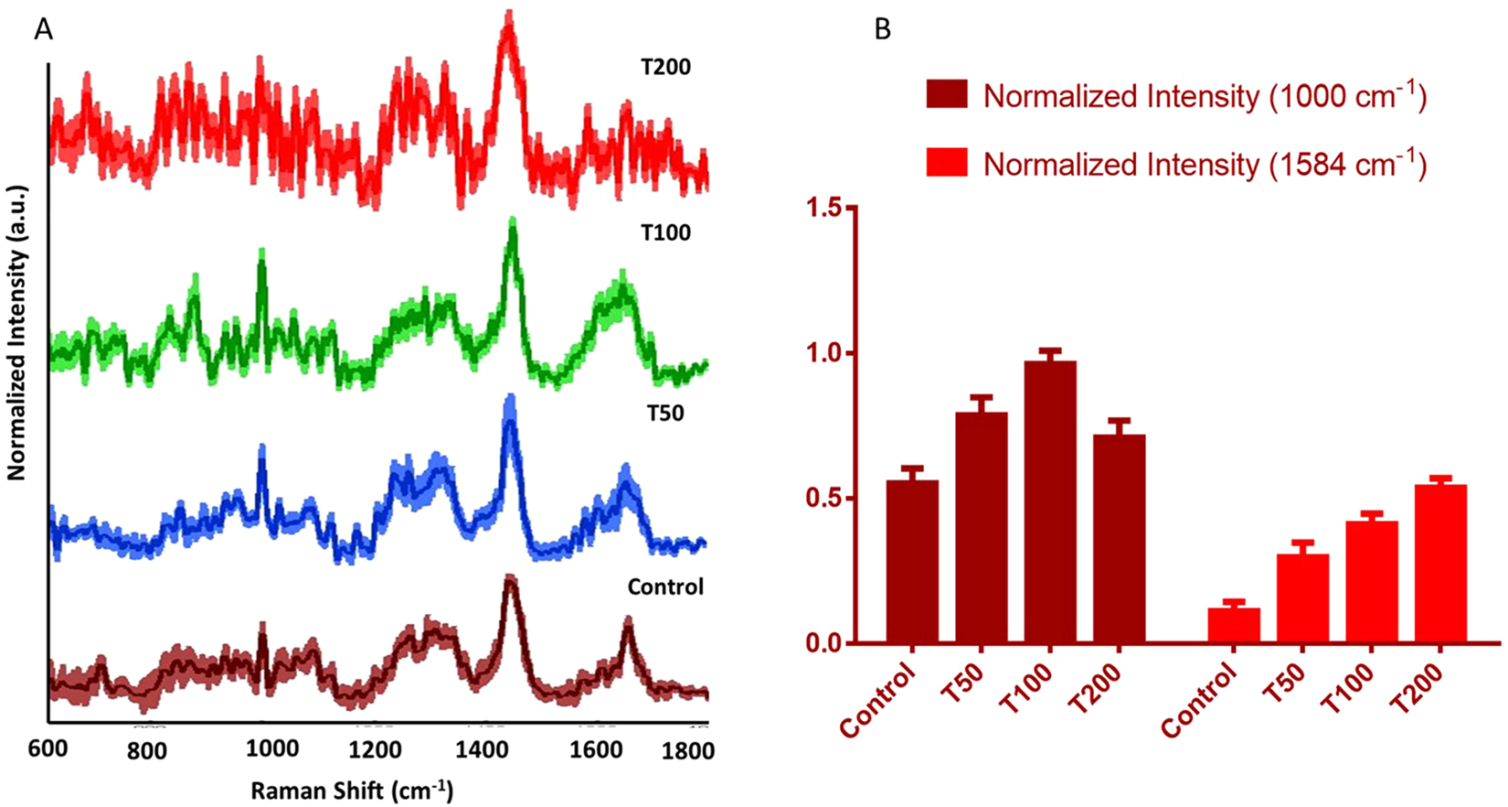
(**A**) Mean baseline corrected spectra along with standard deviation acquired from single cell. Spectra are vertically offset for better visibility. (**B**) Normalized average intensity of 1000 and 1584 cm^−1^ bands along with standard deviations.

Mean baseline corrected spectra along with the standard deviations obtained from single cell are shown in Fig. 2. Spectra acquired from unexposed cells are dominated by typical cellular features indicated by nucleic acids (720, 1093, 1340, 1576 cm^−1^) and protein Raman bands (1004 cm^−1^, amide III and amide I). Sharp band around 1440 cm^−1^ indicates δCH_2_ bands of lipids/proteins. Structural changes in the two major biochemical constituent’s lipids and proteins due UVR exposure is indicated by deformation of amide I (1660 cm^−1^), amide III, tyrosine (835 cm^−1^) and 1440 cm^−1^ bands. Recent studies have shown that changes in bands around 1000 cm^−1^ (attributed to ring breathing vibration of phenylalanine) and 1584 cm^−1^ (−N−H bending vibration of guanine or adenine residues within DNA) can be considered as markers for UVR induced apoptosis in the cell^31, 32^. Corroborating with these results, we also observe enhancement in the intensities of these two bands with exposure time, suggesting activation of apoptotic pathways due to UVR exposure. To quantify these changes, mean scattering intensity for these bands was computed and is presented in Fig. 2B. Clear enhancement in the intensity of 1584 cm^−1^ band with increasing UVR dose was observed. In case of the 1000 cm^−1^ band, enhanced intensity was observed for T50 and T100 cells with respect to controls, as expected. The reduced intensity of the 1000 cm^−1^ band in T200 group can be primarily attributed to the low signal to noise ratio (SNR) due to potential loss of architectural arrangements and resultant disturbance in biochemical composition of cells.

**Figure 3:**
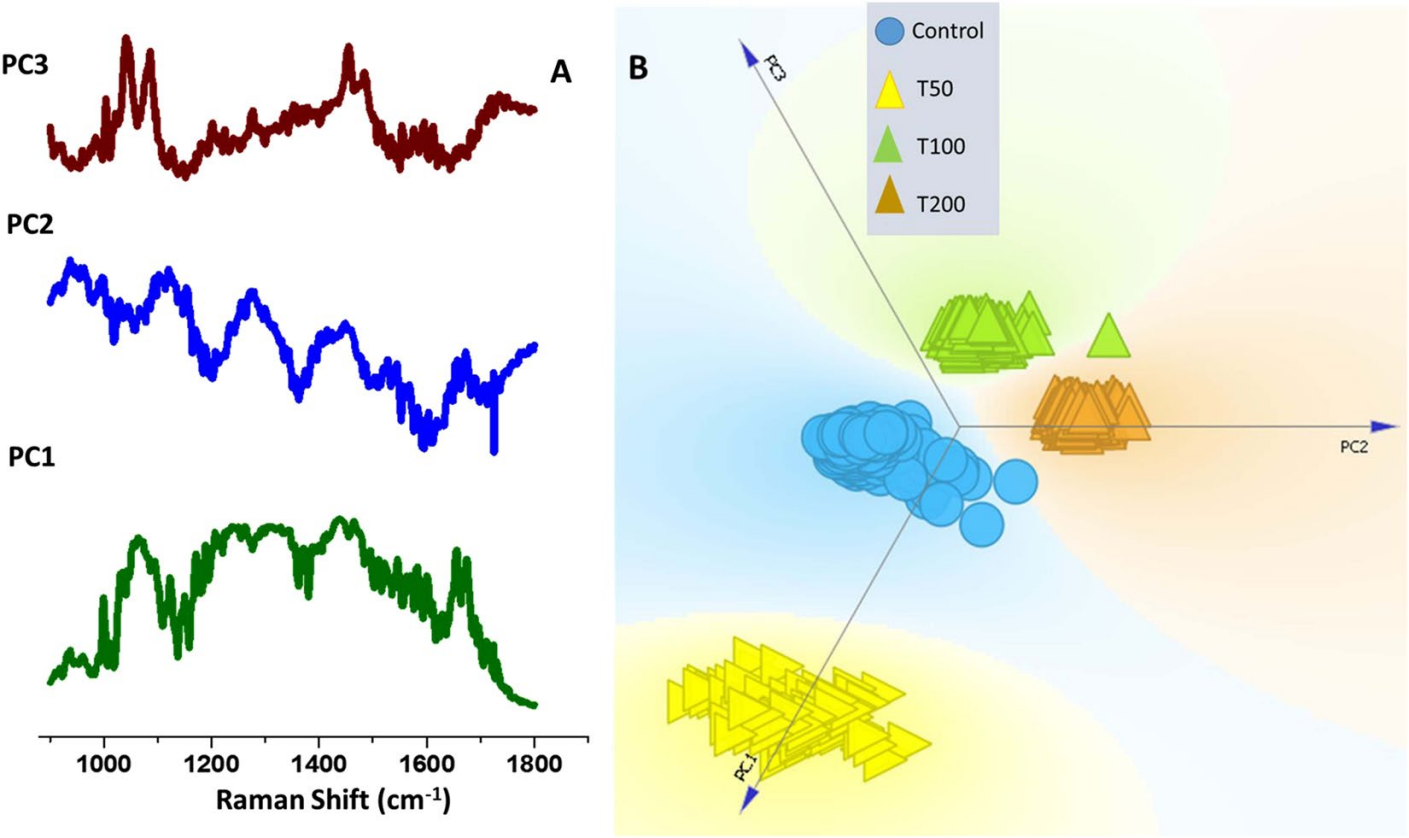
Principal Component Analysis (PCA) of control and UVR treated cells. (**A**) Plot of PC loadings (**B**). Scatter plot of PC scores.

To investigate the consistent differences induced by UVR exposure and, thus, to explore the feasibility of discrimination between exposed and control cells, principal component analysis (PCA) was employed. In Fig. 3A, loading plots of the first three factors with maximum variance contribution are presented. Three dimensional cluster scatter plots generated by the score of these PCs offer visual representation of the inter vs intra-group clustering behaviour (Fig. 3B). Clear separation among control and UVR exposed cells is observed suggesting inherent differences in the spectral (and therefore molecular) profiles of the cells exposed to varying degrees of UVR.

### Phase imaging and biophysical parameters

**Figure 4:**
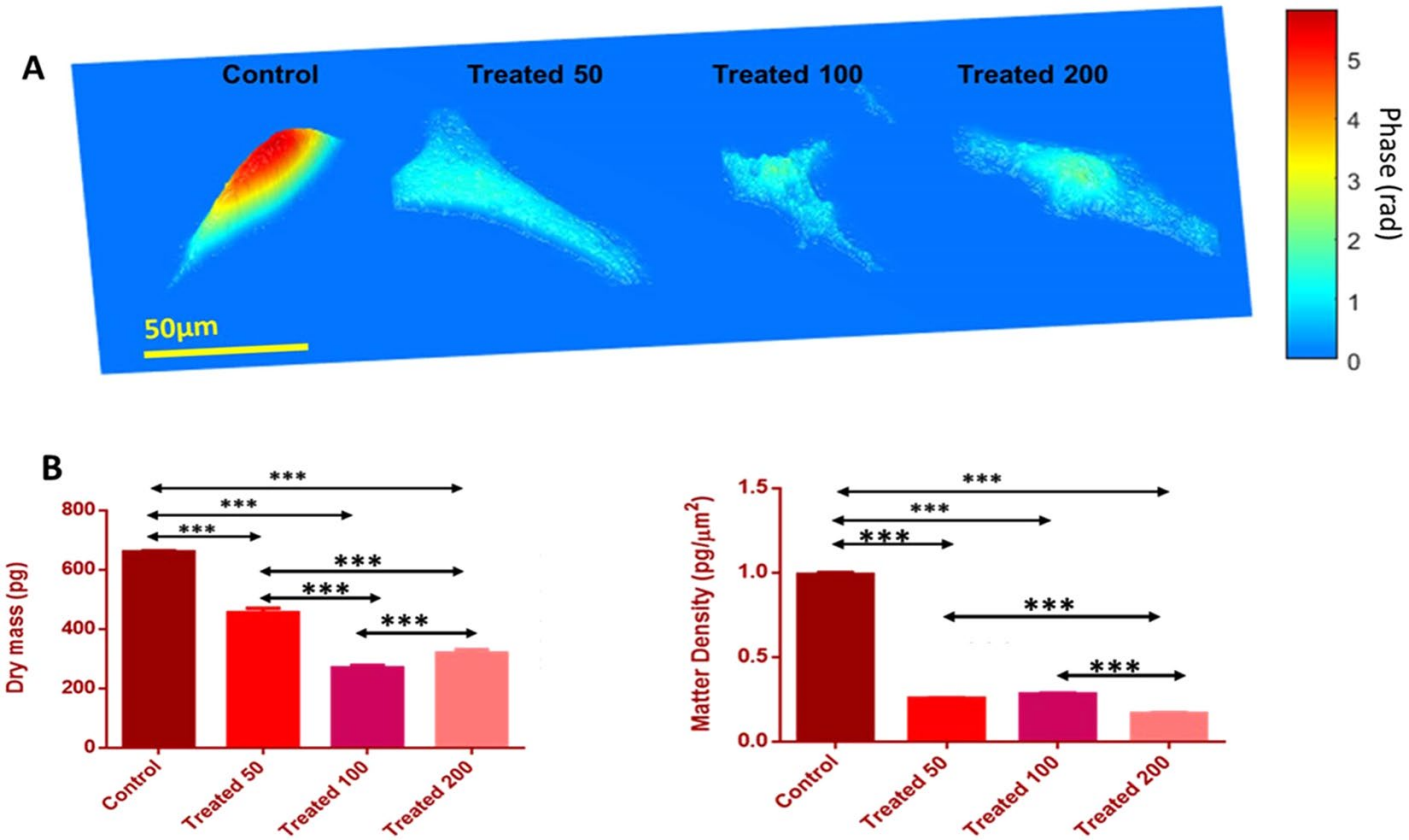
(**A**) Representative quantitative phase images for control and UVR treated skin fibroblasts cells. (**B**) Biophysical parameters calculated using quantitative phase imaging of control and UVR treated cells. Average values along with standard deviation are shown. Unpaired student ‘t-test’ coupled with Welch correction suggest statistically significance differences (***p < 0.0001).

Representative quantitative phase diagrams of cells for control and time bound UVR exposed groups are presented in Fig. 4A. Measured phase maps were used to generate 3D surface images; the color scale corresponds to the phase in radian. Furthermore, the measured phase images were unwrapped and subtracted by the background phase. Changes in terms of dry mass, and matter density of cells were calculated from these phase images to quantify the visible differences. As can be seen from Fig. 4B, significant differences in these biophysical properties among the UVR exposed cells and controls were noted.

## Discussion

Our present study aims at identifying changes in the structural and biochemical composition of skin cells in response to minimal and time-bound UVR exposure in a label-free manner. Previous studies have shown that free radical generation due to photosensitization of riboflavin in the culture medium could induce unwanted phototoxicity to the cells^1, 33^. This can lead to erroneous measurement of actual effects due to radiation damage. Phenol red in the culture medium is useful in quenching this toxicity to some extent but not completely^33^. In order to avoid any discrepancy in the cytotoxic effects of UVR on skin fibroblasts due to culture medium, we have replaced it by phosphate buffer saline (PBS) for time-bound UVR exposure. As mentioned, the effect of UV exposure on cells occurs through the generation of photoproducts or free-radicals. They interfere with replication and other vital cellular machinery ultimately causing cell death. A cytotoxicity assay can provide information about metabolic activity of cells under the influence of external perturbation. In contrast to commonly used 3-(4,5-dimethylthiazol-2-yl)-2,5-diphenyl-tetrazolium bromide (MTT) assay, resazurin dye used in the present study is nontoxic to cells and does not necessitate end point measurements by killing of the cells^30^. By subtracting the absorbance of the blank (culture medium) from the test samples a measure of cellular activity can be obtained. Nonviable cells do not have any metabolic activity so they cannot reduce the indicator dye and therefore time-bound decrease in absorbance of the ‘resorufin’ pigment is observed (Fig. 1).

The studied cells were synchronized to G1 cell-cycle phase to ensure similar biochemical environment for an accurate estimation of changes induced exclusively by UVR exposure. Expectedly, bulk of UVR induced damages operates through generation of free-radicals affecting lipids, proteins and generating double strand breaks or photoproducts affecting the nucleic acid material^27^. As shown in Fig. 2, major changes were observed in the nucleic acid and protein component of the cells. Identification of these changes in a label-free manner on live cells can provide holistic overview about affected cellular machinery. However, it is important to explore if these differences can be utilized to develop a classification model that can be translated into an automated cell pathology recognition system. We have, therefore, also explored the feasibility of discrimination among control and UVR exposed cells using PCA. A number of machine learning algorithms such as linear discriminant analysis, support vector machine, least square analysis have been employed for discrimination of Raman spectral data^34^. Among these, PCA offers a preferred approach for data reduction and exploration. It works by transformation of the data to a new space with linear unrelated variables, i.e. PCs. Utilizing only the first three factors, clear discrimination among exposed and unexposed cells were observed in the PC scores plot in Fig. 3. The corresponding PC loadings provides insights into the spectral features responsible for classification. Consistent with previously mentioned differences in the spectral profiles of control and treated group, changes in nucleic acid, CH_2_ stretch, phenylalanine and amide-I appear to the major discriminatory features in these PCs. These findings suggest that RS in combination with multivariate analysis can be used for objective and label-free biochemical profiling of cells in response to UVR (and likely other external perturbations).

Furthermore, we have shown the promise of QPI in identification of such cellular alterations by leveraging the intrinsic optical contrast generated by phase delay. Biophysical parameters such as matter density and dry mass offer valuable differentiation capability by informing on the morphological changes induced by UVR. Specifically, decrease in cell dry mass and matter density due to UVR treatment indicates disturbances in the structural arrangement. Dry mass is the non-aqueous component of the cells (mostly proteins) and can be mathematically computed by analyzing the measured relative delay maps. As the value is independent of the water content, it serves as a useful parameter to analyze cell response to growth, division and other external influences^35^. As shown in Fig. 4B, decrease in dry mass and matter density with increasing UVR was observed. We attribute the small increase in the average dry mass value of T200 group with respect to T100 to the previously mentioned UVR induced disruption in the architectural arrangement, causing accumulation of intra-/extra- cellular debris and diffused cellular boundary. This makes it difficult to accurately estimate the phase value inside the cell, which may have led to a slight increase in the dry mass for T200 group. However, the values are significantly less than that of the control and T50 groups. Our future validation study will include additional time points to investigate this issue. Overall, changes in dry mass and matter density that are indicators of reduction in protein content and distribution inside the cell is in agreement with RS findings, suggesting its potential utility as complementary markers for estimating UVR induced damages.

While this proof-of-concept study is limited by the relatively small number of cells, it provides novel insights into the impact of UV irradiation-induced changes in skin fibroblasts. Our future efforts will involve incubator integration in the experimental set-up for acquiring data from larger number and diversity of cells in order to fully capture the mechanistic aspects of UV irradiation using label-free quantitative phase imaging and Raman microspectroscopy. Combination of these two techniques, one suited for detection of subtle morphological/biophysical alterations while the other appropriate for capturing molecular perturbations, offers a unique label-free route for monitoring of cellular responses to external stimuli. Given their differential measurement speeds, automated segmentation of quantitative phase images could be used to select and prioritize the sampling points for Raman microscopy. Importantly, quantitative spectral and biophysical measurements provide unique and complementary markers and, as shown in this article, enable objective recognition of distinct radiobiological responses. In addition to revealing radiation-related Raman signatures, our observations provide the first evidence of decrease in dry mass and matter density as potential markers for monitoring radiation exposure in a dose-dependent manner. We envision that the non-perturbing nature of analysis as well as the capability to provide information without the use of contrast agents will be attractive for augmenting biomolecular and phenotyping studies.

## Materials and Methods

### Cell Culture

Normal skin fibroblasts cells (Hs 895.Sk, ATCC Cat# CRL77636) were obtained from the High Throughput Sciences Facility’s cell line repository at the Koch Institute for Integrative Cancer Research at Massachusetts Institute of Technology, USA. Cells were maintained in Dulbecco’s Modified Eagle Medium (DMEM Sigma Aldrich, USA) supplemented with 10% FBS (Sigma Aldrich, USA) and 1% antibiotic solution (Sigma Aldrich, USA) at 37 °C and in a 5% CO_2_ atmosphere. Cell synchronization using serum starvation protocol was performed to bring all the cells in to same phase of cells cycle (G1-phase). Briefly, at ~80% confluency the culture medium was replaced with serum-free medium and cells were incubated overnight. UVR exposure was performed in phosphate buffer saline (PBS) solution. Complete medium was again added after UVR treatment. For RS measurements, cells were cultured on a custom-made petri dishes with quartz cover slip bottom (043210-KJ, Alfa Aesar) to minimize the autofluorescence background^4^. For phase imaging, cells were grown in normal petri dishes at ~60–70% confluency to facilitate acquisition over single cell in the imaging field of view.

### Cell Survival Assay

Optimal UV exposure time was determined using CellTiter-Blue® cell viability assay (Promega Corporation, USA). Cells were grown to 70–80% confluency in 100 mm culture plates and synchronized by overnight serum starvation. Viability assay was performed by adding dye in 1:5 ratio to the culture medium. Cells were incubated in the dark for ~4 hours and absorbance was recorded using UV-2401 PC spectrometer (Shimadzu). 570 and 600 nm were chosen as reference wavelengths, and blank samples consisted of only CellTiter-Blue reagent without cells. Culture medium background at 600 nm was subtracted from experimental values at 570 nm and plot of this difference against the UVR exposure time was generated. Three different time points where cells did not have any structural damage but had significant differences in the absorbance value of the reduced dye product were chosen for further experiments.

### Dosimetry

**Figure 5:**
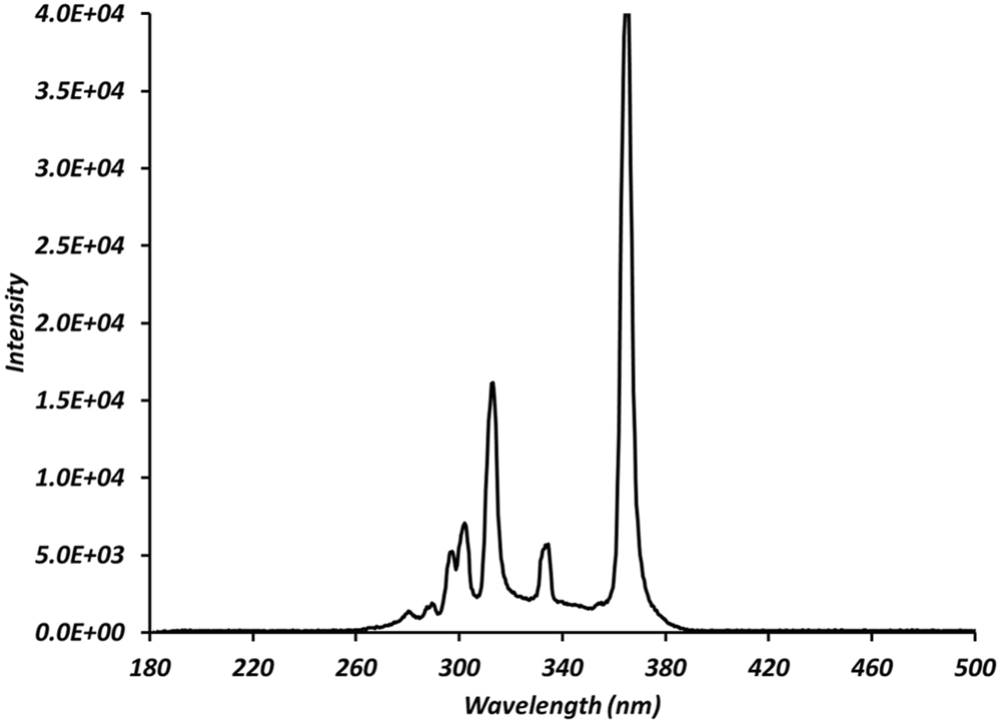
Spectral composition of UV light after passing through applied filters.

For UV irradiation, a mercury lamp (Newport Model Number 66902) was used. A condenser lens was used to focus the light on a band pass filter (FGUV11S, Thorlabs). Spectral output of the system is shown in Fig. 5. Integrating radiation from 280–400 nm yielded a total UV intensity of 1.02 W m^−2^. Three different time points 50, 100 and 200 minutes were chosen to evaluate influence of UVR exposure on cells. At these time points total exposure were at intensities of 0.3, 0.6 and 1.2 J cm^−2^. Once started mercury lamp was allowed to stabilize for 10 minutes. The exposure field of the lamp was sterilized using ethanol. Previous studies have shown that riboflavin in the culture medium can adversely affect the cell survival^1, 33^. In order to reduce photo toxicity by riboflavin of the culture medium, cells in phosphate buffer saline were used for UV treatment. Post exposure complete medium (with serum) was added and cells were incubated for another 24 hours. Control specimens were handled in a similar manner without radiation, and were kept outside the incubator to avoid any differences due to handling.

### Raman spectroscopy

**Figure 6:**
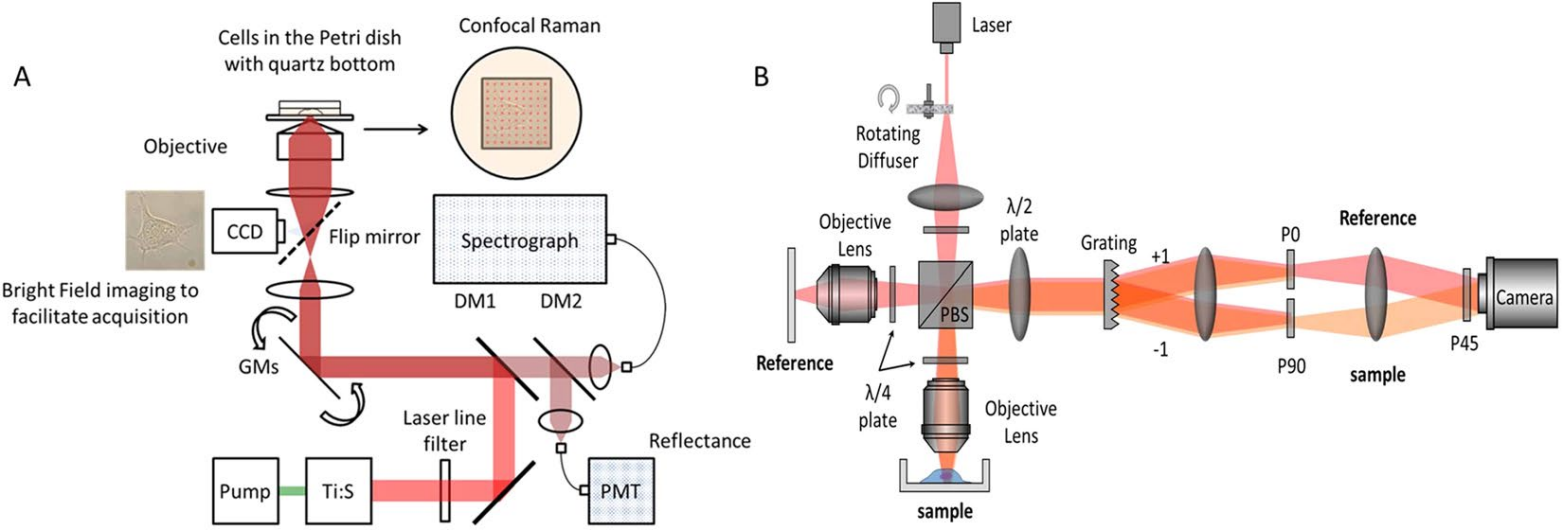
Schematic of (**A**) Raman spectroscopy and (**B**) Quantitative phase microscopy systems employed in the study. DM- dichroic mirror. GM- galvano mirrors.

A previously reported custom-built NIR confocal Raman microscopy system shown in Fig. 6(A) was used for the experiments^3, 36^. Briefly, 785 nm wavelength from Ti: Sapphire laser (3900S, Spectra-Physics) was used as an excitation source. Laser beam was focused on a single cell under the guidance of bright field image. Generated Raman signal was filtered by series of Rayleigh rejection filters and collected by a multimode fiber which is connected to a spectrograph (Holospec f/1.8i, Kaiser Optical Systems) which houses a thermoelectric-cooled, back-illuminated, and deep depleted CCD (PIXIS: 100BR_eXcelon, Princeton Instruments) for spectral measurements. High-speed XY scanning was performed by the galvanometer mirrors (CT-6210, Cambridge Technology). A 1.2 NA water immersion objective lens (UPLSAPO60XWIR 60X/1.20, Olympus) was used to both focus and collect the scattered light with 1 µm spot size. LabView software (National Instruments) and a data acquisition board (PCI-6251, National Instruments) were used to control the system. CMOS camera (BCN-B050-U, MightTex) was also used to capture bright field images. From a single Hs 895.Sk cell, spectra were acquired with an exposure time of 2 seconds at 20 mW/µm^2^ laser power. On average, the total acquisition time for Raman measurements on a single cell was ~10 minutes. Experiments were repeated three times on 5 cells in each group. No visible damage due to laser irradiation was observed.

### Quantitative Phase Imaging

Phase images were acquired by a dynamic speckle-correlation reflection phase microscopy system, which was previously developed in our group and shown in Fig. 6(B) ^37^. Light from a mode-locked Ti:sapphire laser (Mira 900, Coherent) with a centre wavelength of ${\lambda }_{0}=\,800\,nm$ and spectral width Δλ ≈ 17 nm is used. Collimated beam is passed through a rotating glass diffusor (DG1200, Thorlabs), to generate dynamically varying speckle field. The speckle field is split into sample and reference arms of a Linnik interferometer using a polarization beam splitter (PBS). We have put two quarter wave plates for both sample and reference arms to steer the cross-polarized back scattered beams through the output port of the PBS in a collinear fashion. For off-axis holography, a diffraction grating (Ronchi-Ruling, Edmund Optics) was placed in the first conjugate image plane. The 1^st^ and −1^st^ diffracted beams which contains both back-scattered sample and reference beams are then pass through a cross polarizer (P0, and P90). We tuned the orientation of each polarizer such that the sample (reference) beam only pass through 1^st^ (−1^st^) order. Two beams are then combined again at the camera (Flea3, Point Grey) which is placed in the 2^nd^ conjugate image plane. To induce interference between cross-polarized sample, and reference beams, we put another polarizer (45° orientation angle, P45) in front of the camera. With off-axis holographic measurement, we could get single-shot, wide-field quantitative phase images of cells at 100 Hz frame rate. Individual skin fibroblast cells were imaged in the PBS solution using a water immersion objective lens (1.0 NA, 60x, LUMPLFLN 60XW, Olympus). By placing the focus of the objective lens at the bottom surface of the dish, we could get the double-pass transmission phase images of individual cells with high phase-measurement, sensitivity and spatial resolution^37, 38^. For each set of control and exposed group, ~10 cells were selected and 20 phase images were acquired.

### Data Pre-processing and Analysis

Raman spectra were restricted to the fingerprint (600–1800 cm^−1^) region and mean spectrum for each group was generated by averaging over the spectral dataset. Fluorescence background was removed by fitting and then subtracting a 4^th^ order polynomial function. Standard normal variate (SNV) corrections were performed before feeding in to a MATLAB based PCA algorithm. First three factors with maximum variance contribution were chosen to generate a three dimensional scatter plot. Loading vectors of these factors were also generated.

For quantitative phase imaging, the phase $\varphi (x,y)$ of the measured interferograms can be described by following equation

1 $$\varphi (x,y)=2\cdot \int \frac{2\pi }{\lambda }\{{n}_{{\rm{cell}}}(x,y,\,z)-{n}_{{\rm{PBS}}}\}dz$$

where, ${n}_{{\rm{cell}}}(x,y,\,z)$ and ${n}_{{\rm{PBS}}}$ are the refractive indices of the cell and surrounding PBS solution, respectively. The variables $x$ and *y* represent lateral coordinates while $z$ stand for coordinate along the optical axis. The multiplying factor 2 in front of the integration is due to the measurement in double-pass transmission configuration. By integrating the phase over the cell surface area, we can get the dry mass *m* of the cell as:

2 $$m=\frac{1}{\alpha }{\iint }_{S}\frac{\lambda }{4\pi }\varphi (x,y)dxdy$$

where $\alpha$ is the specific refractive index increment, which typically has a value of 0.185 ± 0.002 ${{\rm{\mu }}{\rm{m}}}^{3}/\mathrm{pg}$ for typical cells^23, 35, 39–43^. Subsequently, the mater density of cells is obtained by normalizing the cell dry mass with the area of corresponding cells. For our measurement, we were able to compute the area of individual cells by thresholding the phase value larger than 0.2 radian. Unpaired student ‘t’ test coupled with Welch correction was performed to determine significance of the difference.
